# PROTOCOL: School Absenteeism Among Children and Young People With Disabilities: A Systematic Review

**DOI:** 10.1002/cl2.70033

**Published:** 2025-03-18

**Authors:** Lara Stauvermann, Meike Rau, Vivian Meyer, Isabella Sasso, Michael Feldhaus, Karsten Speck

**Affiliations:** ^1^ Department of Special Needs Education and Rehabilitation University of Oldenburg Oldenburg Germany; ^2^ Institute of Social Sciences, University of Oldenburg Oldenburg Germany; ^3^ Department of Educational Sciences University of Oldenburg Oldenburg Germany

**Keywords:** disabilities, impairment, inclusive education, school absenteeism, school non‐attendance, special education

## Abstract

The systematic review set out in this protocol aims to identify factors influencing school absenteeism among children and young people with disabilities. The focus is on individual, family, and school‐related factors that contribute to absenteeism. The analysis will be based on a qualitative synthesis of the extracted data and findings from the included empirical studies. To achieve this, the review will incorporate qualitative, quantitative, and mixed‐methods studies.

## 1 Background

### 1.1 The Problem, Condition, or Issue

Regular school attendance by children and young people is not only a basic prerequisite for success at school but also for entering the labor market, and thus, it prepares young people for their future lives. Therefore, it can be assumed that, on the one hand, absence from school is associated with significant developmental risks. On the other hand, school absenteeism correlates with many other risk factors (Gren‐Landell et al. 2015; Lenzen et al. 2016; Ricking and Hagen 2016).

Currently, there is no consistent definition of school absenteeism (Kearney 2008; Ricking et al. 2016). Although school absenteeism can be defined in general terms as unauthorized absences from school (Ricking and Hagen 2016), neither the various forms of absenteeism nor their frequencies are clearly defined. School absenteeism appears in various forms. Heyne et al. (2019) define four “school attendance problems.” School refusal refers to staying out of school and avoiding classes due to emotional distress or anxiety. Usually, the school avoiding behavior is known to the parents. In the case of truancy, students stay away from school at irregular intervals without excuse and usually without their parents' knowledge. Heyne et al. (2019) characterize school withdrawal as another form of school absenteeism. It is a form in which the parent or guardian is significantly involved in the child's school absences and either initiates or tolerates the absent behavior. Furthermore, school exclusion means that the student is excluded from school based on a disciplinary action.

In addition to the four types of school absenteeism defined by Heyne et al. (2019), absenteeism is often associated with early dropout, in which the student leaves school early without graduating (Kearney 2008; Ricking 2009). School absenteeism thus increases the risk for early school dropout (Ricking 2009). Therefore, cases of dropout and school exclusion are also considered and included in the review.

Current studies, for example, in Germany, show that the proportion of students leaving school without a qualification is increasing. While in 2013, the total was still 5.7%, the percentage rose to almost 7% (Autorengruppe Bildungsberichterstattung 2020). As a result, approximately 54.000 young people left general education schools in 2018 without a degree. Of these, almost half (44%) of the dropouts are due to students with disabilities (SWD) (Autorengruppe Bildungsberichterstattung 2020). School absenteeism is also a growing problem in the U.S. education system. According to the National Center for Rural Education Research Networks (NCRERN) students were absent between 9 and 11 days each year (National Center for Rural Education Research Networks 2022). In addition, statistics in England show that about 15% of secondary school students and about 11% of primary school students missed 10% or more of possible classes (Department for Education 2021).

Furthermore, several studies show that SWD have significantly higher absenteeism rates compared to students without disabilities (Department for Education 2019). Also, Melvin et al. (2019) identified disabilities as a risk factor for school absenteeism.

The review, therefore, provides an important starting point for further research that will enable recommendations for action to reduce absenteeism in this target group.

### 1.2 The Relationship Between School Absenteeism and Different Types of Disabilities

Previous studies have shown that SWD are more likely to be absent from school (see Chapter 1.1). In addition to the prevalence of absenteeism among SWD, Lereya et al. (2022) also determined differences among different disabilities or impairments in relation to absent behavior. They found that adolescents with physical and motor impairments had the highest rates of absenteeism. Students with emotional and social impairments or learning impairments were also more likely to be absent than young people with other impairments. Chen et al. (2016) examined different categories of impairment related to school absenteeism in a longitudinal study in the United States. The results of the study show that students with severe emotional disturbances and students with learning impairments had an increased risk of chronic absenteeism. Similar findings were shown in a study by Gottfried et al. (2019), which revealed extremely high rates of school absenteeism, particularly among students with emotional disturbances. Children with learning disabilities also had higher rates of absenteeism, according to Gottfried et al. (2019). Results from a survey by Haaland (2017) showed that students with emotional impairments were two and a half times more likely to drop out of school. A German study by Lenzen et al. (2013) also found a correlation in terms of absenteeism and children with behavioral problems.

In this context, the question is why SWD are more likely to be absent from school and what factors might cause this.

Melvin et al. (2019) categorized risk influences for school absenteeism into different dimensions, such as individual, family, school, and social factors (Gren‐Landell et al. 2015; Melvin et al. 2019). This is based on Bronfenbrenner's social ecological theory, which identifies all four categories as important factors in a child's development. As with Bronfenbrenner (1977), the risk factors from the four domains can interact and influence each other. For example, individual factors may include age, gender, or disabilities; school factors may include relationships with teachers; family risk factors may include low family socioeconomic status or family conflicts; and social factors also influence school absenteeism (Bronfenbrenner and Morris 2006; Finning et al. 2019; Gubbels et al. 2019). The aim of this review is, therefore, to identify which factors are associated with school absenteeism in children and young people with disabilities and what impact they have.

### 1.3 Why It Is Important to Do the Review

As already outlined in the previous section, various factors have an influence on school absenteeism (Gubbels et al. 2019; Ricking and Dunkake 2017; Ricking et al. 2009), but a specific consideration for the different contextual factors and causes that increasingly lead to school absenteeism among SWD has not been done yet. There are a few reviews that focus on dropout for SWD (Foreman‐Murray et al. 2022), in distinction, we include different types of school absences. Furthermore, Foreman‐Murray et al. (2022) focus on school engagement and restrictive educational placement, which are only two sub‐areas of school dropout. This review focuses on a broader range of different aspects of school absenteeism.

As in the meta‐analysis by Gubbels et al. (2019), the conducted review aims to identify reasons for school absenteeism. In contrast to Gubbels et al. (2019), the focus in the current review lies on SWD.

The methodological approach in the current review is to include both quantitative and qualitative studies as well as mixed method studies. In addition to many reviews on the topic of school absenteeism, there are no ongoing reviews that consider the topic of school absenteeism among children and young people with disabilities.

## 2 Objectives

This review aims to determine the influence of individual factors, family factors, as well as school factors on the school absenteeism of children and young people with disabilities. On an overarching level, therefore, the question arises what factors impact school absenteeism among children and young people with disabilities. Further differentiated, the following subordinate questions occur:

1. What impact have individual factors on school absenteeism among children and young people with disabilities?
2. What impact do family circumstances have on school absenteeism among children and young people with disabilities?
3. What impact have school‐related factors on school absenteeism among children and young people with disabilities?

## 3 Methods

### 3.1 Criteria for Considering Studies for This Review

The PICOS framework (Methley et al. 2014) was used for developing criteria for this review and has been adapted for the aims of this review. Therefore, a comparison was not considered. In addition, interventions were not explicitly reviewed. Instead, risk and influencing factors at the family, individual, and school levels are examined.

The study types, participants, and types of outcome to be included in this review are described below.

#### 3.1.1 Types of Studies

Eligible study designs that will be considered for inclusion are any type of empirical studies that deal with the topic of school absenteeism among SWD. However, the focus is on the use of primary literature, which is why systematic reviews are excluded.

1. Quantitative studies with various study designs and methods. These may include, for example, cross‐sectional, longitudinal studies, controlled trials, intervention studies, retrospective studies, observational studies, or other designs.
2. Qualitative studies will include the experiences, views or opinions of students, caregivers, or professionals involved in school absenteeism among SWD.
3. Mixed Method studies that combine quantitative and qualitative methods.

#### 3.1.2 Types of Participants

The target group is children and young people with disabilities who are absent from school in any form. With regard to the age group, people up to the age of 25 are included, based on current definitions in England and Germany. Teachers, parents, or other caregivers of an absent student with disabilities can also be considered as participants. For a concrete definition of disabilities used in this study, please refer to Section 3.1.4.

#### 3.1.3 Types of Outcome Measures

##### 3.1.3.1 Primary Outcomes/Critical Outcomes

This review primarily wishes to investigate the current state of research on school absenteeism among children and young people with disabilities in different countries. We expect to investigate different factors that impact different types of school absenteeism among the target group. Since school absenteeism is multifactorial, we assume influences at different system levels (Melvin et al. 2019). Included papers must examine how at least one factor, either on the individual, family, or school‐level, affects student absences.

##### 3.1.3.2 Secondary Outcomes/Other Important Outcomes

Secondary outcomes include every other important information, which is related to school absenteeism and children or young people with disabilities or their caregivers. This could be, for example, interventions or factors contributing to reintegration.

#### 3.1.4 Predictive/Risk Factor: Disability Status

This review's definition of disabilities is oriented to the Individuals with Disabilities Education Act (IDEA), the federal law regulating special education in the United States. The IDEA covers 13 categories of disability that qualify a student for special education. At least one category must be present for a student to receive special education services (IDEA 2018).

1. Autism Spectrum Disorder.
2. Deaf‐Blindness.
3. Deafness.
4. Emotional Disturbance (various mental health problems fall under this category; for the current systematic review, chronic emotional disorders, e.g., Chronic Major Depressive Disorders, Chronic Anxiety Disorders, or Schizophrenia, are included in this category).
5. Hearing Impairment.
6. Intellectual Disability.
7. Multiple Disabilities.
8. Orthopedic Impairment (in this systematic review, students with severe orthopedic impairments or chronic conditions that require special education services, such as cerebral palsy, are included).
9. Other Health Impairment (for this review, this category includes students with chronic diseases that require special education services, e.g., ADHD or Multiple sclerosis).
10. Specific Learning Disability.
11. Speech or Language Impairment.
12. Traumatic Brain Injury.
13. Visual Impairment, including Blindness.

If the study did not explicitly address specific disabilities, studies were included in which a prolonged or severe impairment was assumed. Moreover, papers that report on partial samples are only included if there are specific results for partial samples or subgroups with disabilities.

### 3.2 Search Methods for Identification of Studies

After completion of the pilot search and the finalization of the search string, we conducted a systematic search of all retrievable studies. To ensure the quality of the studies, only peer‐reviewed studies were included.

The following search string was used:

“school absen*” OR “school avoid*” OR “school avers*” OR “school refusal*” OR truan* OR “school phobia” OR “school withdrawal” OR “school exclu*” OR “school attend*” OR “school abstinen*” OR dropout OR “school leaving”

AND

“special need*” OR disab* OR disord* OR “special education*” OR impair* OR autis*

OR disease* OR handicap* OR retard* OR “learning diff*”

In addition to the search strategy mentioned above, which can be used universally for all databases, the search strategy is to be supplemented by database‐specific keywords and controlled vocabulary, which are researched further in the individual databases.

Studies that meet the following criteria were included:

– Published after January 2000 (because the number of papers would otherwise no longer be manageable).
– English or German language.
– Empirical study.
– Target group: students (children and young people involved in the school system) with disabilities, their caregivers or professionals, such as school staff, therapists, or others.
– Disabilities must be present (an independent participation in lessons is not possible without support).
– Type of school absenteeism must be named.

The following electronic databases were searched using the search strategy listed above. These platforms and databases have been selected as they provide coverage of journal articles across a range of important disciplines for the topic of this review. In addition, the databases were selected with consideration of the guide to information retrieval for Campbell systematic reviews (Kugley et al. 2017). The title, abstract, and keywords should be searched.

– Web of Science (provider).
– PSYNDEX (provider).
– APA PsycInfo (provider).
– Education Source (EBSCOhost).
– Publicly Available Content Database (ProQuest).
– ASSIA (ProQuest).
– ERIC (ProQuest).
– Sociological Abstracts (ProQuest).
– Sports Medicine & Education Index (ProQuest).
– PTSDpubs (ProQuest).
– Sociological Abstracts (ProQuest).
– Social Service Abstracts (ProQuest).
– PAIS Index (ProQuest).
– Black Studies Center (ProQuest).
– Worldwide Political Science Abstracts (ProQuest).
– Literature Online (ProQuest).

Because the number of papers would otherwise become unmanageable and the quality of the studies to be included should be as high as possible, gray literature, dissertations, conference proceedings, government documents, and websites of specific education/absenteeism organizations were not included in the work. In addition, neither a hand search of journals nor a search via Google Scholar nor contacting experts in the field was implemented.

### 3.3 Data Collection and Analysis

#### 3.3.1 Selection of Studies

In the screening process, the inclusion criteria (see Section 3.2) are the same for the abstract screening as well as for the full‐text screening. Both processes will be executed using EPPI‐Reviewer. In the abstract screening, four authors are involved, working independently. Approximately 13% of the abstracts will be screened independently but compared afterward to get a good Cohen's Kappa. Two authors will screen the full texts. In this process, the authors will screen ca. 10% independently but compared afterward to get a good Cohen's Kappa. In case of discrepancies during the abstract screening as well as the full‐text screening, the respective studies will be intensively discussed with all authors. As soon as a good Cohen's Kappa is achieved, the authors will continue to work individually and independently. If studies are classified as “unclear” during the further independent screening process, these studies will be subsequently discussed with the other authors.

#### 3.3.2 Data Extraction and Management

Four authors will be involved in the coding process. It is planned that each study will be coded by at least two authors independently using an Excel spreadsheet. The results of the coding process as well as discrepancies will be discussed by the review team. For each study, the following data will be extracted: author names, publication year, journal, country, aim of the study, data type, design, collection method, analysis method, sample size, age, gender, school setting, and further information about the participants as well as the type of disability. Due to the fact that this systematic review is not based on an intervention, but rather is focusing an explicit qualitative synthesis, the outcome will be coded very extensively and dedicated including other aspects: instrument to assess the types of disabilities, definition used of school absenteeism, operationalization of school absenteeism, instrument to assess the frequency and characteristics of school absenteeism, type of disability influencing school absenteeism, and other reasons for school absenteeism. If additional influencing factors (“other reasons”) related to school absenteeism are mentioned (at least three times), they will be added to the coding form. For a better impression of the coding categories see Appendix S1A.

#### 3.3.3 Quality Assessment

Assuming that our review includes both quantitative and qualitative studies and addresses the question of the meaning of school absenteeism among children with disabilities, we are using QuADS (Harrison et al. 2021) as the quality assessment tool. This tool was especially created to rate the methodological and reporting quality in systematic reviews of mixed‐ or multi‐method studies (ibid.). The appropriate items have been added to the full‐text coding form in Appendix S1A. Moreover, the use of QuADS is explained via a guide and the definition of explicit criteria.

The assessment for each study will be conducted by at least two authors. Disagreements will be discussed by two authors and, if necessary, by consulting a third author. Studies with very low study quality, based on the results of the quality assessment, will be excluded after prior discussion by the review team.

#### 3.3.4 Confidence in Cumulative Findings

To assess and transparently present the level of confidence in the results of the qualitative synthesis of this review, it is planned to apply the “The Grading of Recommendations, Assessment, Development and Evaluation Confidence in the Evidence from Reviews of Qualitative Research” (GRADE‐CerQual) assessment (Lewin et al. 2018). The application of the GRADE‐CerQual model makes it possible to create a CERQual evidence profile and a CERQual summary of qualitative findings presented in a table (ibid.). Two reviewers will perform the assessment independently. Disagreements will be discussed with a third reviewer.

#### 3.3.5 Qualitative Synthesis

Our analysis is based on a qualitative synthesis of the extracted data and results of the included empirical studies. As described in Section 3.3.2, the coding of the included studies will be carried out in Excel by at least two authors for every study with the full‐text coding form (see Appendix S1). Afterward, the reviewers will compare their coding list and discuss disagreements with a third researcher.

This review aims to identify factors at the individual, family, and school level in children and young people with disabilities who are absent from school. Therefore, the focus is on a descriptive consolidation and qualitative synthesis of the extracted data. Thematically, this will be structured according to the three levels on which the factors influencing school absenteeism are located. This addresses a limitation of previous reviews, as no review to date attempts to answer the above questions with a synthesis of existing results of empirical studies, breaking down the results of the studies according to the three levels (individual, family, school) that influence school absenteeism.

A meta‐analysis was not conducted because the results of the studies are hard to compare statistically because of heterogeneous samples, various data collection instruments (both for the type of disability and school absenteeism), and different data analysis methods or study designs.

## Author Contributions

- Content: Lara Stauvermann, Isabella Sasso, Meike Rau, Vivian Meyer, Karsten Speck, Michael Feldhaus.
- Review methods: Lara Stauvermann, Isabella Sasso, Meike Rau, Vivian Meyer.
- Information retrieval: Lara Stauvermann, Isabella Sasso, Meike Rau, Vivian Meyer.

## Conflicts of Interest

The authors declare no conflicts of interest.

## Preliminary Timeframe

We plan to submit the completed Systematic Review by December 2025. The search and retrieval process will conclude by June 2025 with submission for peer‐review by December 2025.

## Plans for Updating This Review

There are no plans for updating this review at the moment.

## Sources of Support

### Internal Sources

The authors have nothing to report.

### External Sources

The authors have nothing to report.

## Supporting information

Supporting information.
